# Winemaking Characteristics of Red‐Fleshed Dragon Fruit from Three Locations in Guizhou Province, China

**DOI:** 10.1002/fsn3.2196

**Published:** 2021-03-17

**Authors:** Zhi‐Hai Yu, Jin‐Qiang Li, Shu‐Cheng He, Xian‐Can Zhou, Jia‐Sheng Wu, Qing Wang, Ming‐Zheng Huang, Xiao‐Zhu Liu, Xiao‐Hui Liu, Xun Gong, Wei‐Yuan Tang, Cun‐Bin Xu, Xiao‐Lin Jiang, William James Hardie

**Affiliations:** ^1^ College of Food & Pharmaceutical Engineering Guizhou Institute of Technology Guiyang China; ^2^ School of Food Science and Technology Changzhou University Changzhou China

**Keywords:** dragon fruit wine, geographic influence, harvest month, pitaya, winemaking characteristics

## Abstract

The aim of this study was to identify the locations and harvest months in Guizhou province, China, producing the most suitable red dragon fruit (*Hylocereus polyrhizus*) for winemaking. Fruit from Guanling, Luodian and Zhenfeng counties was harvested separately from successive fruit cycles in August, September and October, respectively. The key traits measured were fruit weight, pulp yield, soluble solids content, and titratable acid. Wine characteristics measured were alcohol content, total carbohydrates, titratable acidity, volatile acidity, and betacyanin content. The overall suitability of fruit from each location for winemaking was evaluated using a multi‐factor, unweighted, scorecard. On that basis, fruit from Guanling county harvested in August was the most suitable. Fruit from Luodian, and Zhenfeng was most suitable when harvested in August and September, and September, respectively. These results provide a preliminary guide for the sourcing of red dragon fruit from Guizhou for wine production.

## Graphical abstract

Identify the locations and harvest months in Guizhou province, China, producing the most suitable red dragon fruit (*Hylocereus polyrhizus*) for winemaking. Fruit from Guanling County harvested in August was the most suitable.

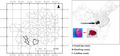

## 1 INTRODUCTION

Guizhou province, located in the southwest of China, is characterized by typical karst landforms (Cai *et al*., 2014) and has a subtropical, humid, monsoon climate with an annual average temperature of 14°C–16°C. However, the low‐altitude valleys of the Hongshui, South Pan (NanPanjiang), and North Pan (BeiPanjiang) rivers, located between 24°37′–25°47′N and 104°31′–107°04′E, occupy a warm‐hot zone where the cumulative temperature ≥10°C reaches more than 5,000°C (Cen *et al*., 2015) and is especially suitable for growing dragon fruit (*Hylocereus spp,* syn. pitaya).

**FIGURE 1:**
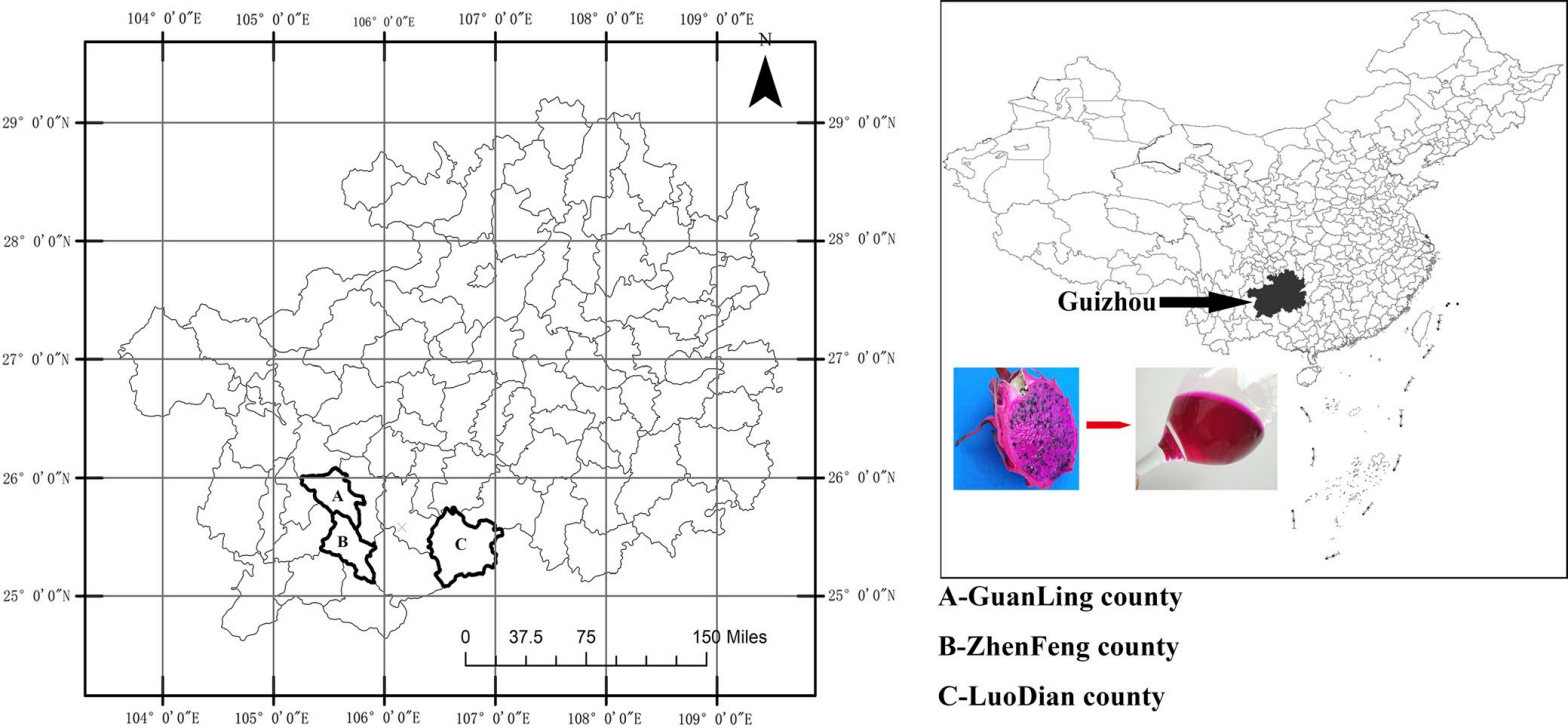
Location of the study areas, Guanling County, Luodian County, and Zhenfeng County in Guizhou province, China

Dragon fruit is an important fruit cultivated in tropical and subtropical areas (Wu *et al*., 2019) and is harvested several times a year; in the northern hemisphere from June to November. Because it is rich in attractive, red‐purple betalain pigments, which have antioxidant properties (Polturak & Aharoni, 2019), the red‐fleshed dragon fruit (*H. polyrhizus*) draws attention from food and pharmaceutical industries. Dragon fruit has been grown in Guizhou since about 2000. Three counties, namely Guanling, Luodian, and Zhenfeng, along the aforementioned valleys, are the major planting locations (Figure 1) and “Zihonglong” is the main cultivar (Wu *et al*., 2019). By 2019, the total area planted to dragon fruit in the three counties exceeded 670 hectares (11089 acres) with an annual output of more than 7,000 tonnes. Totally, the planted area of Guizhou exceeds 6,670 hectares (110,388 acres) with an annual output of more than 50,000 tonnes, placing Guizhou within China's top three provinces for dragon fruit production.

In Guizhou, recent research interest in dragon fruit has mainly focused on the molecular mechanisms of plant abiotic stress tolerance (Fan *et al*., 2014; Li *et al*., 2019) and betalain metabolism (Wu *et al*., 2019, 2020; Zhou *et al*., 2020). There are few reports concerning processing of downstream products of dragon fruit. In preliminary studies we found that red‐fleshed dragon fruit picked in September from Guanling was suitable for making high‐quality table wine with appealing color, typical aroma, and mellow taste.

However, as dragon fruit is also grown elsewhere in Guizhou province and develops in several, successive 30‐day cycles from August to October, we aimed to determine the most suitable dragon fruit producing locations and harvest months for winemaking. Accordingly, we measured the most important fruit traits and winemaking attributes of red dragon fruit harvested separately in three successive months from three major production areas in Guizhou.

### 1.1 Abbreviations

In figures and tables, a combination of letters and numbers indicates the region and month where the dragon fruit was grown and harvested. The letters “G,” “L,” and “Z” indicate Guanling, Luodian, and Zhenfeng, respectively. The numbers “8,” “9,” and “10” indicate “August,” “September,” and “October,” respectively. The letter “F” indicates “fruit.” For example, “G8F” indicates the dragon fruit harvested in Guanling in August. Similarly, G8 indicates wine fermented from the fruit harvested in August from Guanling.

## 2 MATERIALS AND METHODS

### 2.1 Chemicals

All reagents including sugars (sucrose, glucose) were of analytical grade and were purchased from a local supplier (Reggie Biology) in Guiyang, China.

### 2.2 Fruit

Dragon fruit (*H. polyrhizus*, cv. ‘Zihonglong’) used in this study was from Guanling (25°19′–26°05′N, 105°15′–105°49′E, 105°15′–105°49′E), Luodian (25°04′–25°45′N, 106°23′–107°03′E), and Zhenfeng (25°07′–25°44′N, 105°25′–105°56′E) (Figure 1). In each location during the 2017 harvest season (August to October), a total of 27 fruits at commercial maturity, based on external color and size uniformity, were randomly harvested from fruiting cycles maturing in August, September and October. After each harvest, the fruit was randomly grouped into three replicates of nine and manually peeled. Before peeling, the fruit traits were measured. Only the pulp was used for winemaking. After determination of the titratable acid (TA) and soluble solids content (SSC), the remaining must was fermented. Three replicates were used for all chemical analyses.

### 2.3 Fruit traits

Fruit weight (A) and peel weight (B) were recorded for each fruit before peeling, and the pulp yield (C) was determined by the formula, C = (A − B)/A × 100%.

SSC and TA were determined according to the methods described by Zheng *et al*. (2016). For SSC, pulp juice was placed on a digital refractometer (Atago PAL‐1, Tokyo, Japan) and the Brix value was recorded. For the measurement of TA, the pulp juice mixture was filtered with muslin cloth. Ten milliliters of juice was diluted to 100 ml with distilled water and transferred into a 250 ml beaker, which was placed on a magnetic stirrer and stirred continuously. A pH probe was immersed in the solution, and 0.1 M NaOH was slowly added until the pH of the solution reached 8.1. TA was expressed as grams of malic acid per liter of juice (g/L).

### 2.4 Winemaking

A 2 L flask fitted with a water‐filled air lock was used for fermentation. The loading volume was about 75% of the flask capacity. After overnight treatment with pectinase (Lafazyme CL, Laffort, France), 20 mg/L, the sugar content of fruit must was adjusted to approximately 264 g/L with crystalline sucrose, and the pH to 3.6 with tartaric acid. Potassium metabisulphite, 50 mg/L, was added to prevent microbial spoilage, then the must was inoculated with *Saccharomyces cerevisiae* yeast, Zymaflore X16 (Laffort, France). The fermentation temperature was maintained at 16°C–18°C. After alcoholic fermentation (around 15 days), the wine was separated from fermentation lees and settled at 4°C. During settling, the wine was racked from the residue four times. After about six months, the wine was chemically analyzed.

### 2.5 Wine analysis

#### 2.5.1 Determination of total carbohydrate content

The total carbohydrate content of the wine was determined by anthrone colorimetry using 1 ml samples added to 10 ml of 0.1% anthrone solution, comprising 0.1 g anthrone and 1.0 thiourea in 100 ml of 72% H_2_SO_4_, and the absorbance was measured at 620 nm. A calibration curve was prepared as follows; first, 0.0 , 0.2 , 0.4 , 0.6 , 0.8, 1.0 , and 1.2 ml aliquots of glucose standard solution (1.0 mg/ml) were placed in separate tubes and each made up to 2 ml with distilled water. Then, 10 ml of 0.1% anthrone solution, comprising 0.1 g anthrone and 1.0 g thiourea in 100 ml of 72% H_2_SO_4_, was added to each tube, quickly shaken and then placed in a boiling water bath and heated for 10 min. The tubes were then cooled to room temperature and placed in the dark for 10 min. The glucose‐free solution was used as the control. The absorbance of each tube was measured at 620 nm. All tests were performed in triplicate. The calibration was established with the absorbance as the ordinate and the glucose concentration as the abscissa and the equation of the curve was used to calculate the total carbohydrate content of the sample expressed as g/L.

#### 2.5.2 Determination of alcohol content

Alcohol content of the wine was determined according to the national standard GB 5009.225‐2016 published by the Standardization Administration of China (SAC), using the GC method described by Lu *et al*. (2020) with minor modification comprising a column temperature of 157℃ and split ratio 20:1. GC analysis was performed with a Trace 1310 chromatograph (Thermo Fisher Scientific, USA) with flame ionization detector and a TG‐WAXMS column (60 m × 0.25 mm × 0.25 μm). The result was expressed as alcoholic content (%vol).

#### 2.5.3 Determination of titratable acidity and volatile acidity

Titratable acid concentration was determined according to the SAC national standard GB/T15038‐2006, using potentiometric titration as described by Liu *et al*. (2015). TA was expressed as grams of malic acid per liter of wine. Volatile acidity was also determined according to GB/T15038‐2006 using titration with sodium hydroxide after distillation. Volatile acid content was expressed as grams of acetic acid per liter of wine.

#### 2.5.4 Determination of betacyanin content

The content of betacyanin in dragon fruit wine was measured using the spectrometric method described by Tumbas Saponjac *et al*. (2016). The wavelengths 537 nm and 600 nm was used for betacyanin detection, and correction, respectively. Phosphate buffer was used as the blank. Absorbances of betanin were calculated using the following equation:

$$x=1.095\times \left(a‐b\right)$$

where a is absorbance at 537 nm, b is absorbance at 600 nm, and *x* is absorbance of betanin corrected for colored impurities. Betanin in wine was calculated using the equation:

$$C\left(\text{mg}/100\;\text{mL}\right)=x\times F\times 1000/A^{1\%}$$

where *F* is dilution factor and *A* ^1%^ is absorbance coefficient (1,120 for betanin). The betacyanin content was expressed as milligram betanin equivalents per liter of sample.

### 2.6 Determination of fruit suitability for winemaking

Overall fruit suitability for winemaking was determined from a multi‐factor scorecard based on the analytical data. In the absence of further information regarding their relative impacts, individual parameters received no differential weighting (Table 1).

**TABLE 1:** Scoring standards for traits of red‐fleshed dragon fruit for winemaking and the resultant

Items	Range		
Fruit			
Fruit weight (g)	200–250	251–300	301–350
Score	1	2	3
Pulp yield (%)	65–70	71–75	76–80
Score	1	2	3
SSC	10–11.2	11.3–12.4	12.5–13.6
Score	1	2	3
Titratable acidity (g/L)	1.0–1.5	1.6–2.0	2.1–2.6
Score	1	2	3
Wine			
Total carbohydrate (g/L)	≤4	4.1–12	≥12.1
Score	3	2	1
Alcohol (% vol)	8–10	10–12	12–14
Score	1	2	3
Titratable acidity (g/L)	9–20	21–30	31–40
Score	3	2	1
Volatile acidity (g/L)	0–0.6	0.61–1.2	>1.2
Score	3	2	0
Betacyanin (mg/L)	10–20	21–30	>31
Score	1	2	3

### 2.7 Statistical analysis

All data was expressed as the mean ± standard deviation of three replicates. Statistical analysis was performed using SPSS v 23.0 software (SPSS Inc., Chicago, IL, USA). Significant differences among the samples were calculated using one‐way ANOVA followed by Tukey's comparison tests at the 5% level (*p* <.05).

## 3 RESULTS

### 3.1 Key traits of dragon fruit for winemaking

The key wine‐related traits of dragon fruit harvested in three successive months in the three major production locations in Guizhou are shown in Table 2.

**TABLE 2:** The fruit traits

Fruit	Fruit weight (g)	Pulp yield (%)	SSC (%)	TA (g/L)
G8F	327.46 ± 31.12^ad^	76.06 ± 4.00^ab^	13.13 ± 0.17^a^	2.06 ± 0.15^bc^
G9F	264.73 ± 46.77^b^	71.00 ± 11.00^bcd^	13.30 ± 0.26^a^	1.47 ± 0.12^cd^
G10F	293.55 ± 32.50^bc^	78.00 ± 4.00^a^	11.12 ± 0.75cd	1.18 ± 0.14^d^
L8F	290.00 ± 34.35^bc^	67.00 ± 5.00^d^	13.18 ± 0.23^a^	2.26 ± 0.04^ab^
L9F	302.87 ± 29.24cd	71.00 ± 4.00cd	11.77 ± 0.47^bc^	1.79 ± 0.20^c^
L10F	338.74 ± 69.81cd	73.00 ± 4.00^bc^	10.21 ± 0.31^d^	2.59 ± 0.30^a^
Z8F	294.71 ± 35.76^c^	68.00 ± 5.00^d^	11.46 ± 0.31^c^	2.15 ± 0.13^b^
Z9F	296.83 ± 22.26^c^	71.00 ± 3.00^bc^	12.30 ± 0.43^b^	1.88 ± 0.35^bc^
Z10F	324.00 ± 16.94^d^	70.00 ± 5.00cd	12.01 ± 0.21^b^	2.57 ± 0.22^a^

Note

The presented values are mean ± standard deviation (*SD*) of *N* = 27 observations. Different lowercase letters (*p* <.05) indicate significant differences between means.

#### 3.1.1 Fruit weight

Over the three harvest months, the mean weight of fruits ranged from 264.73  to 338.74 g, within which the fruit from Guanling ranged from 264.73  to 327.46 g, from Luodian, 290.00  to 338.74 g, and from Zhenfeng, 294.71  to 324.00 g. In each location, across the three harvest months, the mean weight of fruit from Luodian and Zhenfeng, 310.61 and 304.91 g, respectivey, was markedly higher than that from Guanling, 295.25 g. The weights of Guanling fruit harvested in August, Luodian fruit harvested in September and October, and Zhenfeng fruit harvested in October were significantly higher than those of the others but there were no significant differences within that group.

#### 3.1.2 Pulp yield

Overall, pulp yield ranged from 67 to 78% within which the pulp yield from Guanling, Luodian and Zhenfeng fruit ranged from 71 to 78%, 67 to 73% and 68 to 71%, respectively. The mean pulp yield of fruit from Guanling (75%) was significantly higher than that of fruit from both Luodian (70%) and Zhenfeng (70%).

The pulp yields of Guanling harvested in August and October were significantly higher than the others.

#### 3.1.3 Soluble solids content (SSC)

Overall, SSCs ranged from 10.21 to 13.3%, within which the SSC of fruit from Guanling, Luodian and Zhenfeng ranged from 11.12 to 13.30, 10.21 to 13.18, and 11.46 to 12.30%, respectively. The mean SSC of fruit from Guanling (12.5%) was significantly higher than that of fruit from Luodian (11.72%) and Zhenfeng (11.9%).

Generally, SSCs in August and September were higher than in October, except for fruit from Zhenfeng in which SSC in September and October was higher than in August.

#### 3.1.4 Titratable acidity (TA)

Overall, The TA of the fruit ranged from 1.18  to 2.59 g/L (malic acid equivalents). The TA of fruit from Guanling, Luodian and Zhenfeng ranged from 1.18  to 2.07, 1.79  to 2.59, and 1.88  to 2.57 g/L, respectively. Overall, the mean TA of fruit from Luodian (2.21 g/L) and Zhenfeng (2.20 g/L) was significantly higher than that from Guanling (1.57 g/L). The TA of Zhenfeng and Luodian fruit harvested in October was significantly higher than that of the other fruit with the exception of Luodian fruit harvested in August.

### 3.2 Wine characteristics

Key indicators of the quality of the finished dragon fruit wine, including alcohol, total carbohydrate content, titratable acidity and volatile acidity, and betacyanin content were as follows:

**FIGURE 2:**
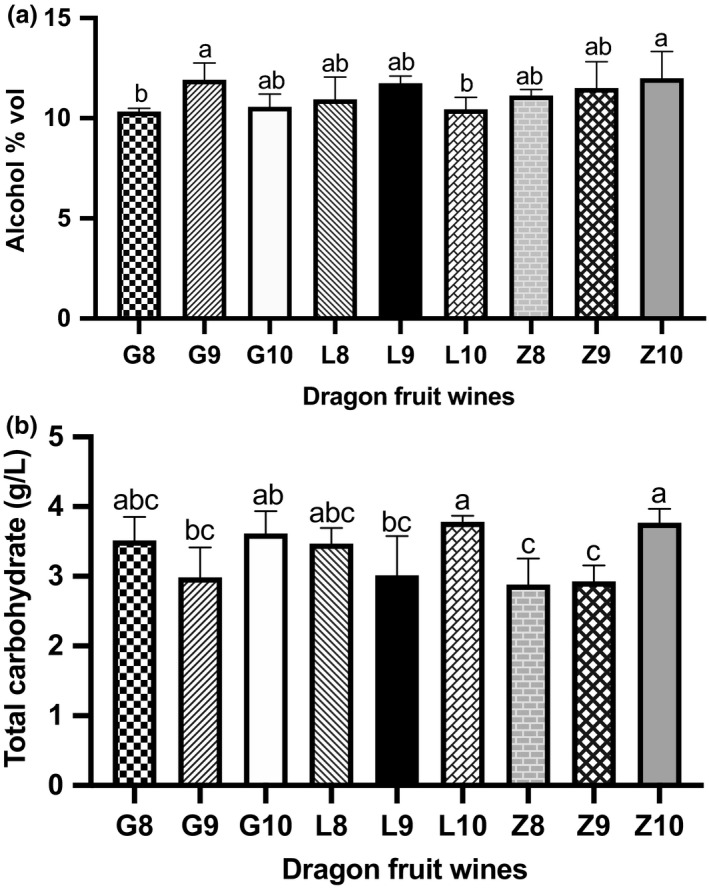
Alcohol and total carbohydrate content. The presented values are mean ± standard deviation (*SD*) of triplicate samples. Different lowercase letters indicate significant differences between means (*p* <.05)

#### 3.2.1 Alcohol

Overall, the alcohol content of the wines ranged from 10.3 to 12% vol (Figure 2a). The alcohol contents of wines from Guanling fruit harvested in September and Zhenfeng fruit harvested in October were significantly higher than those of Guanling and Luodian fruit harvested in August and October, respectively.

#### 3.2.2 Total carbohydrate

The residual carbohydrate content of all wines was lower than 4 g/L (Figure 2b).

#### 3.2.3 Titratable acid

**FIGURE 3:**
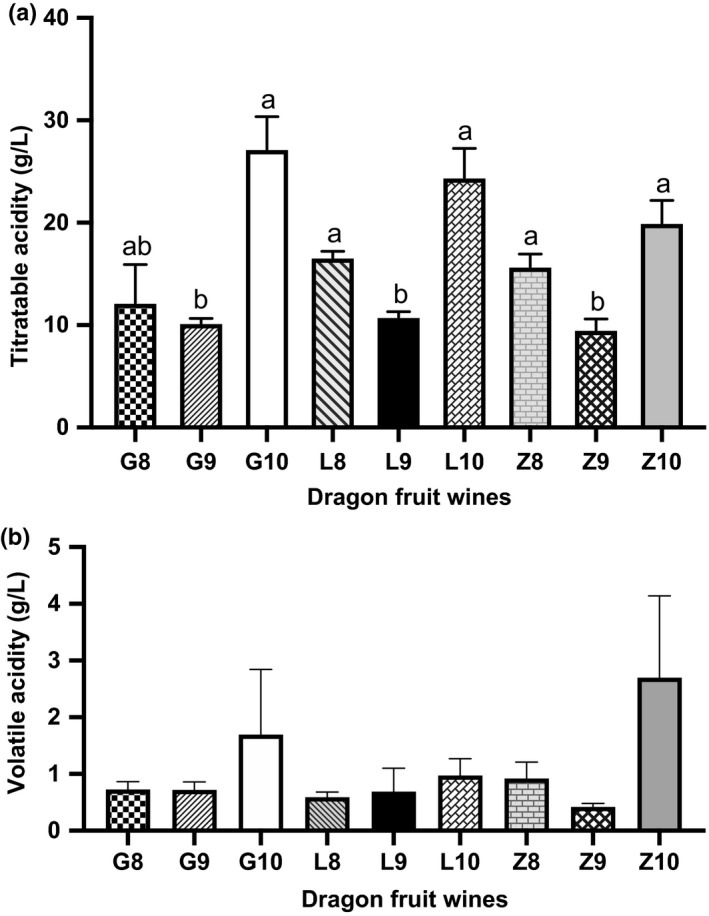
Titratable acid and volatile acid content. The presented values are mean ± standard deviation (*SD*) of triplicate samples. Different lowercase letters indicate significant differences between means (*p* <.05)

The titratable acidity of the wine made from each region was highest from fruit harvested in October (20–27 g/L) and significantly higher than that from fruit harvested in September (Ca 10 g/L) (Figure 3a).

#### 3.2.4 Volatile acid

There were no significant differences in volatile acid content of the wines, and with the exception of wines made from fruit harvested in October, all values were less than 1.2 g/L. The volatile acidity of wines made from fruit harvested in October was markedly higher and more variable (as indicated by standard deviation) than the others (Figure 3b).

#### 3.2.5 Betacyanin

**FIGURE 4:**
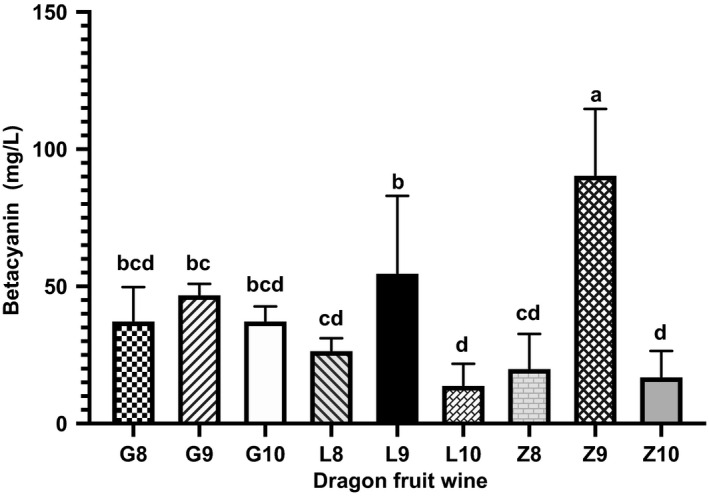
Betacyanin content. The presented values are mean ± standard deviation (*SD*) of triplicate samples. Different lowercase letters indicate significant differences between means (*p* <.05)

The betacyanin contents of wines made from the different locations are shown in Figure 4. Wine from fruit from Zhengfeng had the highest content (90 mg/L) and significantly more betacyanin than the others. In general, within each location, wine made from fruit harvested in September had the highest betacyanin content but the betacyanin contents of each wine made from Guanling fruit were not significantly different.

### 3.3 Scoring of winemaking traits of dragon fruit and wine composition

Based on scores derived from the analytical results and assigned to each fruit batch and the resultant wines (Table 3), fruit from Guanling harvested in August was the most suitable for winemaking, compared to fruit from the other locations. Within the other locations, fruit from Luodian harvested in August and September and fruit from Zhenfeng harvested September, scored most highly.

**TABLE 3:** Score of winemaking traits of dragon fruit and wine

Fruit	Fruit traits				Wine	Wine characteristics					Total score
	Fruit weight (g)	Pulp yield (%)	SSC (%)	Titratable acidity (g/L)		Alcohol (% vol)	Total carbohydrate (g/L)	Titratable acidity (g/L)	Volatile acidity (g/L)	Betacyanin (mg/L)	
G8F	3	3	3	3	G8	2	3	3	2	3	25
G9F	2	2	3	1	G9	2	3	3	2	3	21
G10F	2	3	1	1	G10	2	3	2	0	3	17
L8F	2	1	3	3	L8	2	3	3	3	2	22
L9F	3	2	2	2	L9	2	3	3	2	3	22
L10F	3	2	1	3	L10	2	3	2	2	1	19
Z8F	2	1	2	3	Z8	2	3	3	2	2	20
Z9F	2	2	2	2	Z9	2	3	3	3	3	22
Z10F	3	2	2	3	Z10	2	3	3	0	1	19

## 4 DISCUSSION

The current study, is part of a program that is exploring the potential of red dragon fruit grown in Guizhou for the production of high‐quality wine. The study aimed to identify the most suitable sources of the fruit and the ideal harvest time. Recognizing that wine quality is directly related to the composition of the fruit, our evaluation was based on measurements of fruit, viz. fruit weight, pulp yield, soluble solids content, and titratable acidity, as well as direct analyses of the resultant wine for alcohol content, residual carbohydrate, titratable acidity, volatile acidity and betacyanin.

The range of fruit weight in this study (264‐338 g•FW) was within the previously reported range of red‐fleshed dragon fruit (200–375 g•FW) (Alam Patwary *et al*., 2013). However, pulp yield (67–78%) was lower than that reported in that study (92%). Fruit size may affect the pulp yield through a reduction in the skin to pulp weight ratio as size increases. Notably, we found that, although the fruit from Guanling had the lowest weight, its pulp yield was much higher than that of fruit from the other locations (Table 2). This is likely due to differences in specific skin weight and possibly related to latitude. Guanling is located at a higher latitude than Luodian and Zhenfeng (Figure 1) and we speculate that as result of a latitude‐related factor, *e.g*. temperature or daylength, the skin of Guanling fruit may have been thinner.

During fermentation, *Saccharomyces* yeast converts glucose and fructose into ethanol and CO_2_ (Thesseling *et al*., 2019). However, as dragon fruit, like most other fruits, does not have sufficient glucose or fructose to generate ethanol within the normal wine range, 10‐14%, sugar adjustment of the must, an internationally accepted means of producing fruit wines (Joshi *et al*., 2016), is necessary. Accordingly, the natural sugar content of the juice, as approximated by SSC, is a useful indicator of both fruit ripeness and the required amount of sugar addition. The maximum SSC content of the fruit in this study, 13.3^o^ Brix (or %), was lower than that measured by Nerd *et al*. (1999), 17.5%, in greenhouse‐grown red dragon fruit in Israel. However, in a study of red dragon fruit growing in Malaysia, Sew *et al*. (2013) found that as SSC increased from 11.3 to 12.97^o^ Brix, juice yield increased nearly three‐fold to almost a maximum. While juice yield was not considered in our study, the mean SSCs of fruit from each of our locations were within the seemingly optimal range for that parameter.

The titratable acidity of fruit is an indicator of fruit maturity and potential wine taste. The levels of TA (expressed as malic acid) recorded in this study (1.18–2.59 g/L) were much lower than that of the previously reported range, 3.14–4.8 g/L (Stintzing *et al*., 2003, Sew *et al*., 2013). In order to achieve an acceptable acid and pH balance, the must was acidified before fermentation. Tartaric acid was chosen for TA adjustment because it is one of the most microbiologically stable of the naturally occurring carboxylic acids found in fruit. It also limits the ability of Fe to catalyze wine oxidation (Danilewicz, 2014) and is a widely used additive in the food and beverage industries (Jia *et al*., 2019).

After sugar and acid adjustment, fermentation of all the musts was completed within about 15 days. The alcohol content was within the projected range of 10–12% together with the low residual carbohydrate content indicates complete fermentation of glucose, fructose, and sucrose; the major reducing sugars — both natural and added — present in the must. Thus, indicating the broad suitability of dragon fruit from each location and harvest month for wine production.

Titratable acid present in wine is primarily important for the perception of sour taste (Boulton *et al*., 1999). Changes in TA during fermentation are generally due to changes in the organic acid profile of the must due to depletion (*e.g*., of malate and citrate) and excretion (*e.g*., of succinate, acetate and lactate) which accompanies yeast metabolism. Tartaric acid is not metabolized by wine yeast (Mendes Ferreira and Mendes‐Faia, 2020) but may decrease by precipitation of potassium bitartrate. The large increases in TA, measured after fermentation, from initial fruit contents are largely attributable to the addition of tartaric acid. Similarly, the wide range of wine TA is chiefly attributable to differences in the organic acid contents of the fruit and differences in the amount of tartaric acid adjustment.

The volatile acidity of wine is a measure of the portion of total acidity that is volatile, *viz*. acetic acid and related compounds. In amounts greater than ca 1.2 g/L it is an indicator of microbial spoilage. The high mean level of volatile acidity of wines made from fruit harvested in October is a concern. It is not clear if this was a result of microbial spoilage of the fruit but the high soluble solids content of the fruit, indicates over‐ripeness and that microbial activity was likely.

The betacyanins, (betanin and isobetanin) are red‐violet colored betalain compounds present in red dragon fruit (Choo *et al*., 2018) and which impart their distinctive color and antioxidant properties to wine. The higher concentrations of betacyanin in wine from fruit grown in Zhengfeng and Luodian, both at similar latitudes, together with the observation that in all three locations fruit harvested in September imparted the most betacyanin, strongly indicates a latitude‐related influence of daylength and/or temperature on this feature of the fruit.

With some exceptions due to excessive acidity (titratable and volatile), most wines met analytical criteria of acceptability in terms of alcohol content, residual carbohydrate, titratable acidity, volatile acidity and color. Accordingly, this study confirms that dragon fruit produced in Guizhou is suitable for winemaking and has sound development prospects. Based on our scorecard, fruit harvested in August from Guanling was the most suitable for dragon fruit winemaking. The scorecard approach also indicated the most suitable months of harvest in Luodian and Zhenfeng. However, with extension of similar research over a greater number of seasons and greater understanding of the relative importance of individual parameters in meeting wine quality specifications, and weighting the parameters accordingly, the utility of this approach is likely to be improved.

In the course of this study, we have recognized several technical aspects of dragon fruit winemaking that are worthy of further attention. Two matters seem particularly important, *viz*. the selection of indigenous yeasts, and the instability of betacyanins.

The fermentation of fruit must into wine is an ecologically complex process, in which yeasts play a fundamental role (Suranska *et al*., 2016). In common with most other fruit winemaking, in this study our fermentation relied on yeast originally selected from grape and used predominantly for making white grape wines. Notably, we have found only one report concerning the influence of different yeasts on the physicochemical and oenological properties of red dragon fruit wine (Jiang *et al*., 2020). Certainly, there is no specific yeast isolated from local dragon fruit for wine fermentation. Yet, even in the highly advanced grape winemaking, the systematic selection of indigenous yeasts is an ongoing activity.

The selection of indigenous yeast adapted to survive with dragon fruit, appears particularly important because its fermentation for quality wine production presently relies on yeasts adapted to grape which, apart from having about twice the sugar content, has a distinctly different metabolic profile. Accordingly, systematic selection of yeasts from local dragon fruit plantings on the Yunnan‐Guizhou Plateau represents an opportunity for improved fermentation efficiency and quality of dragon fruit wine at both natural and adjusted must sugar levels.

Potential instability of betacyanins is another issue. Betacyanins, with their attractive color and many health functions (Timoneda *et al*., 2019), are an important feature of red‐fleshed dragon fruit wine. However, direct exposure to light, pH, dissolved oxygen and high temperature can all cause betacyanins to discolor (Amjadi *et al*., 2018; Esatbeyoglu *et al*., 2015). Unless these influences are carefully managed, in our experience, the shelf life of dragon fruit wine is around only two months.

Although the addition of ascorbic acid is reported to stabilize betacyanin content in red‐fleshed dragon fruit (*H. polyrhizus*) juice and concentrate (Wong and Siow *et al*., 2015), we have observed that ascorbic acid added to dragon fruit wine has little effect on the stability of betacyanin during storage at room temperature. Furthermore, while satisfactory stability of betacyanin in a low alcohol (1.5%vol) dragon fruit beverage during eight weeks storage at 4℃ has been demonstrated in a laboratory study (Choo *e* *t al*., 2018), that result is yet to be confirmed in large scale production. Consequently, while the mechanisms behind the instability of betacyanin remain unclear (Kumorkiewicz *et al*., 2019), further study is needed to understand this phenomenon; especially in regard to dragon fruit wine‐related factors.

## 5 CONCLUSIONS

In Guizhou, geographic factors and month of harvest strongly influence key physicochemical attributes of red dragon fruit *H. polyrhizus*, cv. Zihonglong, for winemaking. Based on a multi‐factor, unweighted scorecard, we conclude that within the valleys of the Hongshui, South Pan, and North Pan rivers of south‐western Guizhou province, red dragon fruit grown in Guanling and harvested in August is the most suitable for winemaking. Within Luodian, fruit harvested in August and September is more suitable for winemaking than fruit harvested in November. Within Zhenfeng, fruit harvested in September is the most suitable. However, weighting of individual factors to meet particular wine specifications are likely to improve the utility of this approach. Further study in other seasons will be required to confirm these findings.

## CONFLICT OF INTEREST

All authors declare that there is no conflict of interest.

## AUTHOR'S CONTRIBUTION

Yu Zhi‐Hai, Huang Ming‐Zheng and William James Hardie conceived this project. Yu Zhi‐Hai wrote the manuscript, Li Jin‐Qiang sampled and investigated the agronomic traits of dragon fruits. He Shu‐Cheng, Zhou Xian‐Can, Wu Jia Sheng, and Wang Qing performed the determination experiment for dragon fruit wine. Liu Xiao‐Zhu, Liu Xiao‐Hui, Gong Xun, Tang Wei‐Yuan, Xu Cun‐Bin, and Jiang Xiao‐Lin provided help for evaluation of dragon fruit wine. WJH critically reviewed and improved the manuscript.

## ETHICAL APPROVAL

This article does not contain any studies with human participants or animals performed by any of the authors.

## Data Availability

All data included in this study are available upon request by contact the corresponding author.
